# ProTox: a web server for the *in silico* prediction of rodent oral toxicity

**DOI:** 10.1093/nar/gku401

**Published:** 2014-05-16

**Authors:** Malgorzata N. Drwal, Priyanka Banerjee, Mathias Dunkel, Martin R. Wettig, Robert Preissner

**Affiliations:** 1 Structural Bioinformatics Group, Institute for Physiology, Charité - University Medicine Berlin, Lindenberger Weg 80, 13125 Berlin, Germany; 2 Graduate School of Computational Systems Biology, Humboldt-Universität zu Berlin, Invalidenstraße 42, 10115 Berlin, Germany; 3 German Cancer Consortium (DKTK), Im Neuenheimer Feld 280, 69120 Heidelberg, Germany

## Abstract

Animal trials are currently the major method for determining the possible toxic effects of drug candidates and cosmetics. *In silico* prediction methods represent an alternative approach and aim to rationalize the preclinical drug development, thus enabling the reduction of the associated time, costs and animal experiments. Here, we present ProTox, a web server for the prediction of rodent oral toxicity. The prediction method is based on the analysis of the similarity of compounds with known median lethal doses (LD50) and incorporates the identification of toxic fragments, therefore representing a novel approach in toxicity prediction. In addition, the web server includes an indication of possible toxicity targets which is based on an in-house collection of protein–ligand-based pharmacophore models (‘toxicophores’) for targets associated with adverse drug reactions. The ProTox web server is open to all users and can be accessed without registration at: http://tox.charite.de/tox. The only requirement for the prediction is the two-dimensional structure of the input compounds. All ProTox methods have been evaluated based on a diverse external validation set and displayed strong performance (sensitivity, specificity and precision of 76, 95 and 75%, respectively) and superiority over other toxicity prediction tools, indicating their possible applicability for other compound classes.

## INTRODUCTION

About 100 million rodents are used annually in animal trials, many of them for toxicity testing in the pharmaceutical or cosmetic industry. European regulations require *in vivo* testing of novel industrial chemicals, biocides, pesticides and cosmetic ingredients for acute oral toxicity (1). Similar regulations are in place in other countries, too. However, following the Replace, Reduce, Refine (3Rs) principle (2), the development and optimization of alternative methods including *in vitro* assays and *in silico* predictions have been put into focus in the recent years.

According to estimations, drug candidates entering clinical trials have only an 8% chance of becoming marketed drugs and about 20% of failures in the late drug development are caused by occurrences of toxicities (3,4). Computational predictions offer many advantages in the drug development process. They can be applied early in the drug discovery process and therefore save the amount of time as well as the *in vitro* and *in vivo* experiments required. Predictions of pharmacokinetic properties including the absorption, distribution, metabolism, excretion and toxicity (ADMET) also provide a method to prioritize the selection of compounds to be synthesized and evaluated as drug candidates. Toxicity predictions take place at different levels of complexity, ranging from predictions of toxic endpoints such as acute toxicity or carcinogenicity to predictions of the underlying mechanisms of toxicity development, e.g. the identification of targets involved in adverse drug reactions and toxic effects. Predictions of toxicity endpoints are mainly divided into statistical models and so-called expert systems (5). Whereas expert systems incorporate human knowledge and expert toxicity rules such as structural alerts (4), statistical models include quantitative structure–activity relationships (QSAR) and machine learning algorithms trained on compounds with known toxic activity. On the other hand, target-based toxicity predictions mostly apply methods also used in computer-aided drug design to identify novel hit compounds. In particular, pharmacophore models have emerged as a fast technique which can be easily applied for profiling purposes (6–8).

*In silico* toxicity predictions are integrated into several commercial software environments including Discovery Studio's TOPKAT^®^ (Toxicity Prediction by Komputer Assisted Technology; Accelrys, Inc., USA), ADMET Predictor™ (Simulations Plus, Inc., USA) or ADME-Tox Prediction (Advanced Chemistry Development, Inc., Canada). Freely accessible tools are also available for a variety of endpoints and are mainly based on QSAR models and expert systems. Examples of QSAR-based methods which present prediction of acute oral toxicity include the Toxicity Estimation Software Tools (T.E.S.T.) developed by the U.S. Environmental Protection Agency or the web server AdmetSAR which includes a variety of ADMET prediction models (9). Although QSAR models can show good hit rates in toxicity prediction, it is not clear how they will perform for structurally novel compounds not included in the training set. While the integration of newly available data requires the redevelopment of new QSAR models, other methods like similarity searches in databases are fast and can easily be extended to include new information. Similarity methods are based on the assumption that structurally similar molecules should manifest similar biological activities and many successful applications of similarity-based methods have been reported, including the identification of novel hits for drug discovery, drug repositioning studies or the prediction of drug–target interactions, side-effects and therapeutic indications (10–14). Thus, the use of similarity-based methods might also represent a promising approach for the prediction of toxic effects.

Here, we present ProTox, a web server for the prediction of median oral lethal doses (LD_50_ values) and toxicity classes in rodents. The prediction method is based on the analysis of the two-dimensional (2D) similarity to compounds with known LD_50_ values and the identification of fragments over-represented in toxic compounds. In addition to the oral toxicity prediction, the web server indicates possible toxicity targets based on a collection of protein–ligand-based pharmacophores (‘toxicophores’) and therefore provides suggestions for the mechanism of toxicity development. The prediction methods are fast and the underlying database structure allows simple and fast upgrades of the prediction server when new data becomes available. A validation on an external dataset suggests good performance of ProTox in comparison to several other *in silico* toxicity prediction tools.

## PROTOX INTERFACE

### Input

The ProTox web server has an easy-to-use interface and the only requirement is the 2D structure of the molecule for which the toxicity is to be predicted. The user has the possibility to draw the structure with an embedded chemical editor or use the integrated PubChem search (https://pubchem.ncbi.nlm.nih.gov/) which allows the search for chemical structures using the compound name. Additionally, ProTox allows the upload of files containing one or more compounds in the mol format. This is particularly interesting for early drug discovery projects, where drug candidates could be evaluated using ProTox with respect to their potential toxicities. A possible input for ProTox would be, for example, a hit list obtained from high-throughput or virtual screening and the predicted toxicities could be used for prioritization of compounds for further developments.

### Output

**Figure 1.**
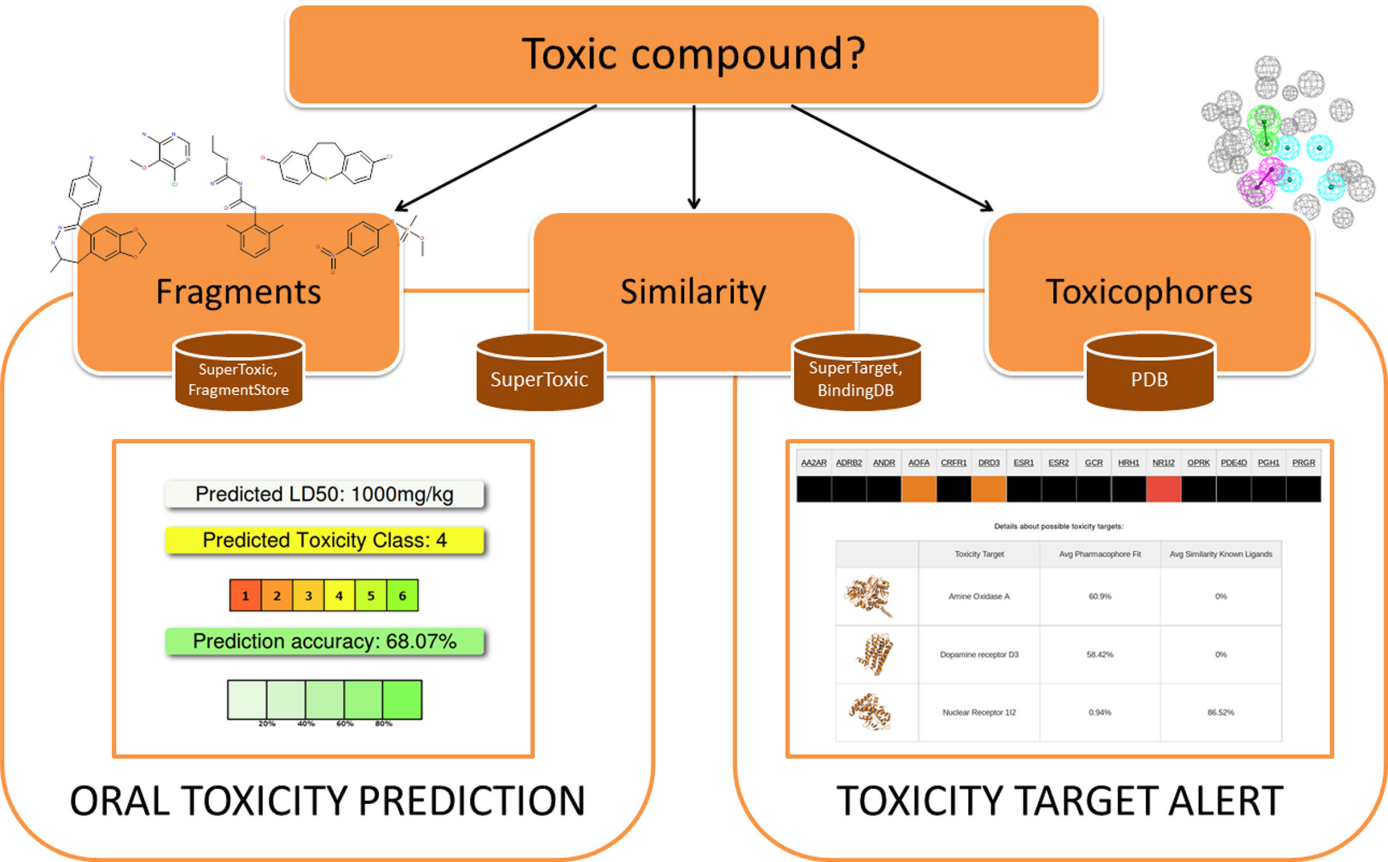
ProTox web server output functionality.

Typically, a toxicity prediction report for one compound is generated within seconds. The report can be divided into two parts: the prediction of the acute oral toxicity and the indication of possible toxicity targets (Figure 1). The oral toxicity prediction results are based on the analysis of 2D similarities and the recognition of toxic fragments, as described below. In addition to the prediction of the LD_50_ in mg/kg, the input compound is classified into a toxicity class ranging from I to VI, according to the globally harmonized system of classification of labelling of chemicals (GHS, United Nations, first revised edition 2005). The prediction accuracy derived from cross-validation results is also given. Additionally, the structure of the input compound as well as its physicochemical properties are displayed. In the similarity section, the three most similar compounds of the database which are used for the prediction of the oral toxicity, their properties and classification are shown. Furthermore, the fragment section indicates if and where toxic fragments occur in the input compound.

In the second part, the possible binding to defined toxicity targets is indicated. Currently, 15 toxicity targets which have been associated with adverse drug reactions are covered, but future updates of the target list are planned. Wherever available, further information about the protein targets is given as links to the SuperTarget (15,16) database. The toxicity target alert is based on ligand mapping to pharmacophore models developed from targets with known experimental structures and the similarity of the input molecule to known ligands of a target, as described below. For every input compound, a toxicity target profile is provided in form of a 4-coloured heat map. The more intense the colour, the higher is the probability of binding. Moreover, details are provided for every indicated toxicity target, including the similarity to known ligands of that target and the average mapping to the toxicophore models.

## PREDICTION METHODS

### Acute oral toxicity prediction

To predict the toxicity of the input compound, a 2D similarity search is performed on an updated version of the in-house toxicity database SuperToxic (17) and the most similar compounds to the input molecule are considered. The set used for prediction consists of approximately 38 000 unique compounds with known oral LD_50_ values measured in rodents. The data was gathered from public sources and literature and prepared using Instant JChem 6.2.0 (January 2014), ChemAxon (http://www.chemaxon.com), for standardization purposes.

From the standardized molecule structures, InChI keys were calculated and used to remove duplicates in the dataset. In the case of multiple LD_50_ values measured for one compound, the lowest dose value was kept to represent the worst-case toxicity of a compound. Six toxicity classes were defined based on the GHS classification scheme using the LD_50_ thresholds of 5, 50, 300, 2000 and 5000 mg/kg body weight. Each compound of the dataset was represented using a concatenated fingerprint consisting of the ‘FP2’ and ‘FP4’ fingerprints of Mychem (http://mychem.sourceforge.net/) as well as the ECFP4 fingerprint (18). The fingerprints were calculated using Open Babel (19) and JChem 6.1.3 (November 2013), ChemAxon (http://www.chemaxon.com), respectively. The similarity between two compounds was calculated using the Tanimoto Index.

In addition to the similarity search, the prediction method takes into account the presence of toxic fragments. All compounds in the database were fragmented using RECAP (20) as well as the in-house method ROTBONDS (21). The occurrence of each distinct fragment in molecules of the prediction dataset was tested using its SMILES string, computed with JChem 6.1.3 (November 2013) in a substructure search which was implemented using Open Babel's (19) fast search. To determine fragments over-represented in the most toxic classes, a propensity analysis (22) was performed. Propensity scores (PS) were calculated for every fragment and toxicity class. Toxic fragments were defined as those showing a PS above a threshold of 3 in classes I, II or III, and a PS below 1 in classes IV–VI. Based on these conditions, a total number of 1591 and 1580 fragments specific to toxicity classes I–III, generated with the ROTBONDS and RECAP fragmentation method, respectively, were contemplated for prediction.

### Indication of toxicity targets

Protein targets involved in toxic and adverse effects, also referred to as toxicity targets, are represented as a set of protein–ligand-based pharmacophore models. Toxicity targets were defined as all targets belonging to the Novartis *in vitro* safety-panel of protein targets associated with adverse drug reactions (23–25). However, future considerations of additional targets such as the Qiagen list of targets involved in molecular responses to toxic drugs (more than 350 targets; http://www.qiagen.com) are planned. From the 73 targets, 15 were found to have at least one experimental structure representing a human protein–ligand complex. All structures were downloaded from the PDB website (26) and a toxicophore was generated for each complex using the active site ligand as input and the Discovery Studio software (version 3.1; Accelrys Inc., USA). Water molecules were kept if present in the structure and the pharmacophore containing all features matching the observed protein–ligand interactions was used for further investigation. In order to allow mappings to compounds other than present in the experimental structure, large pharmacophores containing more than six features were, in general, divided into all possible subsets of 6-feature models. However, an exception were the opioid receptor κ1 (OPRK) pharmacaphores where 5-feature subsets were used in order to obtain predictive pharmacophores (see below) and estrogen receptor 1 (ESR1) pharmacophores where 7-feature subsets were used to reduce the number of combinations.

All toxicophore models which passed an external validation (see below) were mapped to the in-house toxicity database and the fit values indicating the quality of the ligand–pharmacophore fit were extracted. For the indication of possible toxicity targets of an input compounds on the ProTox web server, the three most similar compounds of the in-house dataset identified using the FP24 fingerprint and using a Tanimoto threshold of 0.7 are considered. The average fit value of the reference compounds to all validated pharmacophore models belonging to a toxicity target are displayed in the toxicity report. To support the target indication, all ligands known to bind to the 15 investigated proteins were extracted from BindingDB (27) and filtered to contain only molecules with activity values (IC_50_, EC_50_ or *K*_i_) below 100 μM. The average Tanimoto of the three most similar compounds from this set is combined with the average toxicophore fit to give suggestions about possible toxicity targets.

## VALIDATION

### Optimization of oral toxicity prediction using cross-validation

**Figure 2.**
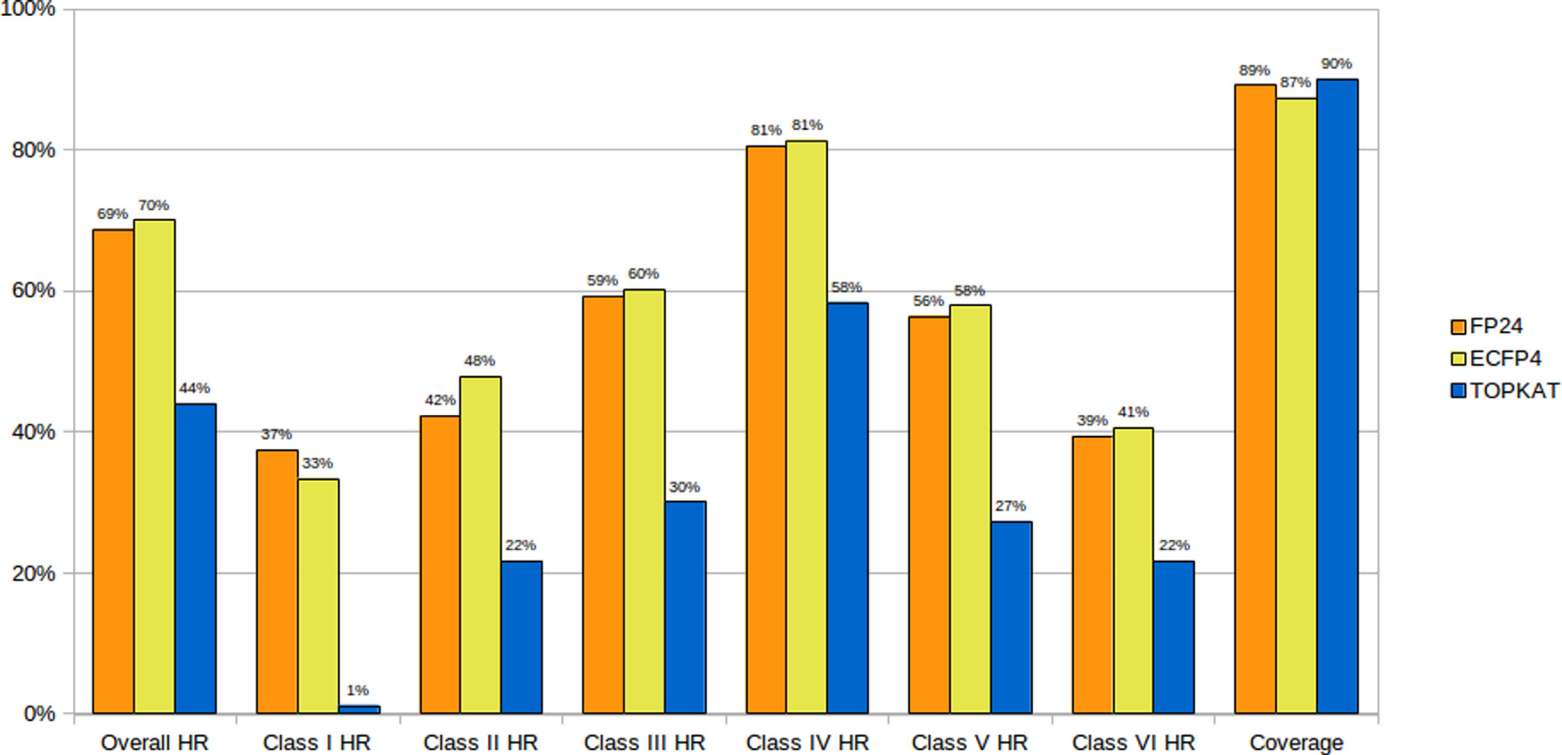
Performance of ProTox in leave-one-out cross-validation compared to TOPKAT^®^. The overall hit rate (HR) as well as the hit rates obtained in the individual toxicity classes are displayed for ProTox using FP24 fingerprints (orange), ECFP4 fingerprints (yellow) and TOPKAT^®^ (blue). Due to the use of Tanimoto similarity thresholds of 0.7 and 0.5 for FP24 and ECFP4, respectively, toxicity predictions were not made for all compounds and the prediction coverage is indicated. In case of TOPKAT^®^, only molecules lying with the optimum prediction space were considered.

To determine the best parameters for the similarity search, a leave-one-out cross-validation was performed on the training set and the results compared to the results of the commercial TOPKAT^®^ software. Different parameters were tested in the similarity search, including the consideration of different fingerprints, multiple nearest neighbors and different measures to combine the toxicity values for multiple compounds (mean, median, minimum, Tanimoto-weighted average). It was observed that TOPKAT^®^ yielded a prediction coverage of 90% when using the rat oral model and considering compounds within the optimum prediction space. A similar coverage was obtained with the ProTox similarity search using a Tanimoto cutoff of 0.7 and 0.5 for the FP24 and ECFP4 fingerprints, respectively (89 and 87%). As indicated in Figure 2, the overall hit rate of TOPKAT^®^ was lower than 50%, with a very low number of hits among the most toxic compounds (class I). On the contrary, all tested parameters of the ProTox similarity search resulted in an overall hit rates of ∼50–75%. A detailed description of the cross-validation results can be found in the Supplementary Tables S1 and S2. The best prediction rates were obtained when combining the LD_50_ values of the three most similar compounds using the median value. The ECFP4 fingerprint resulted in a slightly higher overall hit rate than the FP24 fingerprint (70.05 in comparison to 68.78%), but a lower hit rate among the most toxic compounds (Figure 2) and was therefore not considered further. It was also evaluated whether the combination of oral toxicity class prediction based on 2D similarity and the presence of toxic fragments into a consensus prediction method could improve the performance in the most toxic classes. The calculation of an average toxicity class based on the similarity as well as the presence of fragments over-represented in classes I, II and III indeed resulted in increased hit rates for the most toxic compounds (increase between 3 and 10% for toxicity classes I–III). Therefore, the consensus method combining similarity and toxic fragment searches was implemented in the ProTox web server.

### External validation

In order to evaluate the prediction method, a subset of the SuperToxic database (17) developed previously was chosen as an independent validation set. The validation set represented ∼5% of the dataset used for prediction and exhibited a similar distribution of compounds in different toxicity classes. The performance of ProTox with and without the consideration of toxic fragments was compared to the performance of TOPKAT^®^ and the freely available T.E.S.T. programme. In particular, the sensitivity, specificity and precision were calculated for each toxicity class and method used. An overall sensitivity, specificity and precision for each tool were determined as an average of all six toxicity classes weighted by the number of molecules per toxicity class. For both TOPKAT^®^ and T.E.S.T., the oral rat LD_50_ prediction models were used and in case of T.E.S.T., the nearest neighbour method was chosen which predicts the toxicity based on the activity of closest compounds in the QSAR descriptor space. As shown in Table 1, ProTox showed a similar prediction coverage and specificity to the other programmes tested. However, independently of the method used, ProTox clearly outperformed TOPKAT^®^ and T.E.S.T. by almost 30% regarding the sensitivity and precision for the external validation set used. The consideration of toxic fragments increased the prediction rates for the most toxic classes, in particular the prediction rate for class I molecules by 6%, thus confirming the applicability of the consensus method. In order to make the results more transparent and to allow comparisons of ProTox to other tools, we have made a subset of the validation set available for download from the ProTox website. The performance analysis of the small validation set is summarized in Supplementary Table S3.

**Table 1.** Performance of ProTox on external set compared to other toxicity prediction programmes

	ProTox (FP24)^a^	ProTox (FP24 and fragments)^b^	TOPKAT^®^^c^	T.E.S.T.^d^
Sensitivity (%)	75.56	73.08	44.8	46.27
- Class I	66.67	72.73	0.00	0.00
- Class II	65.52	61.80	1.15	22.37
- Class III	66.79	67.88	29.43	23.33
Specificity (%)	95.11	94.62	88.96	89.25
Precision (%)	75.17	73.50	41.98	45.61
Coverage (%)	90.14	91.78	89.40	78.64

Sensitivity, specificity and precision were calculated as described in the External validation paragraph. Coverage refers to the percentage of compounds for which a prediction could be made.

^a^ProTox similarity search using FP24 fingerprints.

^b^ProTox consensus prediction based on similarity search (FP24 fingerprint) and toxic fragment identification.

^c^TOPKAT^®^ (Accelrys Inc., USA) oral rat LD_50_ model.

^d^T.E.S.T. (USA) oral rat LD_50_ model using nearest neighbour prediction.

### Toxicity target pharmacophore validation

To evaluate the toxicity target pharmacophores, a validation set was created for each target. Active compounds were identified from the SuperTarget database (15,16) as all compounds with a *K*_i_, *K*_d_, IC_50_ or EC_50_ below or equal to 1 nM. For each active compound, up to 10 decoy molecules were property-matched from the all-clean ZINC (recursive acronym for 'ZINC is not commercial') database (28). Similar to other decoy-generating algorithms (29,30), a decoy was accepted if its properties were similar to the active compound (molecular weight ± 25 Da, log *P* ± 1, number of hydrogen bond acceptor ±2, number of hydrogen bond donors ±1, number of rotatable bonds ±1, same total charge). Furthermore, it was required that a decoy must be dissimilar to other decoys for a particular target (Tanimoto < 0.9 calculated using ‘FP2’ fingerprint, see above) as well as dissimilar to all active molecules for this target (Tanimoto < 0.75).

Toxicity target pharmacophores were mapped to the validation sets and the predictive power of toxicophore fit values was evaluated in a receiver-operating characteristic analysis. Only toxicophores resulting in an area-under-the-curve above 0.6 were kept for every target. It should be noted that predictive toxicophores could only be developed for 14 out of the 15 toxicity targets. Details of the pharmacophore validation results are summarized in the Supplementary Table S4.The training set compounds were mapped to the validated toxicophores using Discovery Studio 3.1 (Accelrys Inc., USA) and, in case of multiple pharmacophores for one toxicity target, fit values were combined to an average toxicity target fit.

## CONCLUSIONS AND FUTURE DIRECTIONS

To the best of our knowledge, the ProTox web server is the first freely available toxicity prediction method based on chemical similarity and the identification of toxic fragments and demonstrates good performance in comparison to available QSAR-based methods. A novelty of the ProTox server is the integration of a toxicity class prediction using similarity- and fragment-based methods with alerts of possible toxicity targets, therefore providing insights into the mechanisms involved in toxicity development. A major advantage of ProTox is the capability of future additions which will include newly available toxicity data, the incorporation of other administration routes, endpoints and organisms as well as the addition of other toxicity targets and pharmacophores. In order to keep the web server at a high standard, updates are planned on a quarterly level.

## SUPPLEMENTARY DATA

Supplementary data is available at NAR Online.

Supplementary Data
