# Supplementary material for: ProTox: a web server for the in silico prediction of rodent oral toxicity
Source: Nucleic Acids Res. 2014 May 16;42(Web Server issue):W53–8. doi: 10.1093/nar/gku401 (PMC4086068; doi:10.1093/nar/gku401)
Supplement: Supplementary Data [file supp_42_W1_W53__index.html]

Supplementary Data 

# ProTox: a web server for the *in silico* prediction of rodent oral toxicity

## Supplementary Data

**Files in this Data Supplement:**

- SUPPLEMENTARY DATA
