# Supplementary material for: ProTox: a web server for the in silico prediction of rodent oral toxicity
Source: Nucleic Acids Res. 2014 May 16;42(Web Server issue):W53–8. doi: 10.1093/nar/gku401 (PMC4086068; doi:10.1093/nar/gku401)
Supplement: Supplementary Data [file supp_gku401_nar-00651-web-b-2014-File004.pdf]

SUPPLEMENTARY DATA

S1. Cross-validation results with FP24 fingerprint and Tanimoto cutoff of 0.7

Hit rates using mean dose value of x similar compounds for prediction:

x similar	Overall	Class I	Class II	Class III	Class IV	Class V	Class VI
1	67.05	42.05	45.10	58.66	77.49	54.83	43.26
2	67.86	29.23	37.70	55.06	79.75	57.40	47.11
3	67.61	23.85	33.38	51.48	80.80	57.23	48.26
4	67.57	20.26	33.02	49.67	81.22	57.36	49.76
5	67.39	16.41	32.94	47.91	81.68	56.83	49.76

Hit rates using median dose value of x similar compounds for prediction:

x similar	Overall	Class I	Class II	Class III	Class IV	Class V	Class VI
2	67.86	29.23	37.70	55.06	79.75	57.40	47.11
3	68.78	37.44	42.24	59.23	80.58	56.47	39.35
4	68.90	31.28	40.56	56.28	81.56	57.49	40.37
5	69.31	33.33	42.46	58.13	82.26	56.07	37.00

Hit rates using minimum dose value of x similar compounds for prediction:

x similar	Overall	Class I	Class II	Class III	Class IV	Class V	Class VI
2	65.59	56.67	53.88	65.25	76.04	45.63	26.77
3	62.88	62.56	55.42	66.80	73.25	38.44	19.43
4	60.26	64.87	54.76	66.41	70.41	33.86	15.28
5	58.16	65.64	53.95	66.34	67.84	30.32	14.32

Hit rates using Tanimoto-weighted average dose value of x similar compounds for prediction:

x similar	Overall	Class I	Class II	Class III	Class IV	Class V	Class VI
1	66.87	42.05	45.02	58.47	77.30	54.50	43.44
2	67.31	29.23	37.12	53.74	79.16	57.36	48.01
3	67.06	23.85	33.24	50.33	80.12	57.31	48.74
4	67.07	20.26	33.09	48.38	80.72	57.36	49.88
5	66.87	16.15	32.28	46.39	81.16	57.07	50.18

S2. Cross-validation results with ECFP4 fingerprint and Tanimoto cutoff of 0.5

Hit rates using mean dose value of x similar compounds for prediction:

x similar	Overall	Class I	Class II	Class III	Class IV	Class V	Class VI
1	67.88	40.00	49.43	59.12	78.10	55.54	43.79
2	68.97	28.95	42.37	56.27	80.79	56.96	49.16
3	68.88	20.53	40.47	54.30	81.41	57.28	49.66
4	68.68	20.26	37.89	52.08	81.81	57.52	50.16
5	68.38	18.95	35.46	51.17	81.58	57.98	50.72

Hit rates using median dose value of x similar compounds for prediction:

x similar	Overall	Class I	Class II	Class III	Class IV	Class V	Class VI
2	68.97	28.95	42.37	56.27	80.79	56.96	49.16
3	70.05	33.42	47.91	60.25	81.44	57.96	40.67
4	70.03	28.95	45.25	58.47	82.10	57.86	42.42
5	70.22	30.53	48.75	59.33	82.32	57.05	40.36

Hit rates using minimum dose value of x similar compounds for prediction:

x similar	Overall	Class I	Class II	Class III	Class IV	Class V	Class VI
2	66.46	56.58	54.59	66.10	76.80	46.36	27.39
3	63.77	64.74	55.28	67.05	74.06	39.48	20.77
4	61.68	69.47	54.90	66.81	71.77	35.73	16.97
5	59.85	71.32	54.37	66.49	69.61	32.75	15.47

Hit rates using Tanimoto-weighted average dose value of x similar compounds for prediction:

x similar	Overall	Class I	Class II	Class III	Class IV	Class V	Class VI
1	67.42	40.00	49.20	59.00	77.28	55.79	43.86
2	68.24	28.95	42.14	54.99	79.89	56.90	49.53
3	68.06	20.79	40.70	52.68	80.49	56.84	50.34
4	68.07	20.26	37.97	50.98	81.02	57.52	50.66
5	67.79	18.95	35.76	49.97	80.96	57.64	50.97

S3. Performance analysis of small external validation set

Compound ID	LD50 (mg/kg)	Toxicity class	ProTox – predicted class	Topkat® - predicted class	T.E.S.T. – predicted class
22	100	3	4	3	4
123	837	4		4	4
171	1000	4		4	
200	309	4		4	4
202	2200	5		5	4
307	400	4	4	5	6
379	7.36	2	3	3	5
404	800	4		3	4
484	378	4	3	2	4
539	5000	5	4	5	4
590	1314	4	4	6	4
691	300	3	4	4	4
830	173	3	3	4	4
954	570	4	4	4	4
1080	1000	4	4		3
1116	2260	5	4	5	6
1260	84.94	3	3	4	
1294	570	4	4	4	4
1374	100	3	2		3
1393	1447	4	5	4	5
1411	285	3		3	4
1431	1238	4	4	4	4
1453	180	3		3	
1541	237	3	4	4	
1599	129	3	4	3	4
1677	176	3	3	4	4
1754	2000	4	6		2
1894	7.9	2	2	4	4
2099	4200	5	5	4	6
2227	2300	5	5	4	4
2301	2500	5	5		3
2302	225	3	3	6	5
2364	4228	5	4	4	5
2374	50000	6	5	6	5
2440	200	3	4	6	5
2536	540	4	4	4	3
2648	15000	6	6	6	6
2858	1448	4		4	3
2888	580	4	4		6
3154	460	4		3	4
3160	400	4	3	3	4
3182	54.8	3	3	4	3
3311	300	3	3	4	4
3314	1269	4	6	4	
3320	550	4	4	4	4
3374	1800	4	5	4	4
3440	1020	4	4	6	4
3487	1000	4	4	2	6
3503	4200	5	6	4	5

Compound ID	LD50 (mg/kg)	Toxicity class	ProTox – predicted class	Topkat® - predicted class	T.E.S.T. – predicted class
3564	133	3	3	4	
3579	750	4	3	3	4
3665	1000	4		3	3
3873	1100	4	4	4	5
3938	15000	6	4	3	3
4079	94	3	3	3	3
4190	2.7	1	2	4	2
4231	1100	4	4	4	5
4327	6000	6	5	6	6
4385	700	4	4	4	4
4474	300	3	4	4	4
4593	275	3	3	3	
4647	290	3	4	5	5
4656	2800	5	5	5	6
4763	800	4	4	4	4
4768	380	4	4	3	3
4849	2500	5	5	5	4
4872	926	4		3	
4964	3.8	1	1	2	2
5087	1480	4	4		4
5215	384	4	4	5	4
5492	1500	4	4	6	5
5547	800	4	4	4	5
5551	2000	4		4	4
5564	700	4	4	4	3
5648	6300	6	4	4	4
5783	15600	6	6	6	
5795	475	4	4	4	3
5855	2050	5	5	6	4
5990	2400	5	4	4	4
6344	500	4	4		4
6448	750	4	4	5	5
6479	1700	4	4	4	4
6602	2000	4	4	4	
6772	600	4		4	3
6859	8000	6		6	3
6913	4550	5		4	4
7066	700	4	4	4	4
7164	750	4	4	5	
7213	114	3	3		5
7360	353	4	6	5	5
7425	1000	4		3	5
7451	1200	4	5		4
7835	350	4	4	3	2
7836	253	3	3	4	4
7919	1000	4	4	6	4
7948	780	4	4	3	2
8009	4984	5	3	4	5
8160	350	4	4	5	4
8340	225	3		3	3
8535	300	3		3	4

Compound ID	LD50 (mg/kg)	Toxicity class	ProTox – predicted class	Topkat® - predicted class	T.E.S.T. – predicted class
8656	2000	4		4	3
8754	594	4	4	4	5
8759	1070	4	4	3	1
8778	2000	4	4		3
8949	22000	6		4	
9001	293	3	4	5	4
9073	280	3	4	4	4
9107	1950	4	4	4	4
9123	219	3	3	4	6
9249	700	4	4	4	5
9327	3130	5	4	4	4
9341	476	4		4	4
9934	1000	4	4	5	4
9981	401	4		4	4
9985	800	4	4	4	4
10007	860	4	4		4
10178	200	3	3	4	4
10197	1250	4	4	4	4
10200	2500	5	4	4	4
10220	599	4	1	4	3
10278	1400	4	4	4	4
10287	5000	5	4	6	5
10341	3100	5	5	3	4
10354	1500	4	4	4	4
10367	2000	4	4	4	
10411	2190	5	5	3	3
10699	1519	4	4	6	5
10913	138	3	3	3	3
10947	630	4	4	5	2
10979	1000	4		6	4
10991	1600	4	4	5	4
11088	2197	5	4	5	4
11100	560	4	4	4	4
11120	240	3	3	6	4
11192	1250	4		5	4
11215	2000	4	1		1
11258	2450	5	5	4	5
11517	1000	4	4	5	5
11576	410	4	4	3	5
11664	4000	5	5	5	6
11825	9500	6	4	5	3
11866	4000	5	5		4
11880	305	4	4	3	3
12056	4000	5	2	4	2
12099	17.5	2	2	3	3
12111	1000	4	4	4	4
12132	325	4	4	4	2
12266	1000	4	4	4	5
12307	320	4	4	4	5
12315	5000	5		6	
12400	1300	4	4		4

Compound ID	LD50 (mg/kg)	Toxicity class	ProTox – predicted class	Topkat® - predicted class	T.E.S.T. – predicted class
12489	706	4	4	4	4
12526	121	3	3	4	3
12548	880	4	4	5	5
12659	700	4	4	4	4
12688	100	3	2	2	2
12717	600	4	3	4	5
12783	150	3	3	4	3
12895	202	3		4	
13000	290	3	3	4	4
13121	62	3	3	4	1
13223	1150	4	4	4	5
13416	1000	4	4	4	5
13520	1000	4	4	4	3
13542	650	4	4	4	4
13564	1000	4	4	4	4
13614	1800	4	5	4	6
13631	400	4		4	4
13675	595	4	4	5	4
13727	2000	4	5	5	5
13773	2000	4	4		4
13831	282	3	3	5	4
13890	235	3	4	5	5
13903	282	3	3	3	3
13956	44	2	3	5	4
13972	2000	4	4	4	
13995	262	3	3	4	3
14101	0.45	1	1	3	4
14131	485	4	4	4	4
14173	120	3		4	4
14338	108	3		5	3
14399	200	3	4	5	3
14459	135	3	3		4
14516	4000	5	5	4	
14562	5000	5	6	5	6
14586	3305	5	5	4	
14590	500	4	4	5	5
14600	2500	5		3	
14799	8000	6	6	3	4
14857	70	3	3	4	4
15134	800	4	4	6	5
15143	2000	4	4	4	
15222	1235	4	5	4	4
15344	240	3	4	3	4
15414	580	4	4	3	6
15508	85	3	3	4	4
15585	700	4	4	5	6
16062	9500	6	6	4	5
16099	2190	5	5		5
16143	1110	4	5	4	5
16144	2500	5	5	4	4
16296	1570	4	4	5	4

Compound ID	LD50 (mg/kg)	Toxicity class	ProTox – predicted class	Topkat® - predicted class	T.E.S.T. – predicted class
16415	425	4	3	3	3
16435	450	4	4	3	4
16494	100	3		4	4
16514	205	3	3	6	5
16686	1000	4	6		
16733	2000	4	4	4	5
16956	5100	6	5	4	4
16967	1500	4	4	4	4
16979	1580	4	3	4	
17119	620	4	4	4	4
17131	4000	5	5		6
17169	105	3	3	3	3
17256	750	4	4	3	5
17273	590	4		4	4
17310	3000	5	5	4	4
17378	15000	6	6		6
17397	30	2			
17434	263	3	4	3	4
17741	1810	4		4	6
17757	200	3	3	4	4
17799	388	4	5	3	
17981	500	4	5		3
18060	1125	4	4	4	4
18111	1200	4	3	5	1
18158	800	4	4		4
18222	780	4	4	5	4
18365	7650	6		4	4
18376	2000	4	4	3	4
18491	3000	5	4	3	4
18495	3000	5		3	5
18710	2000	4	4	4	6
18751	375	4	4		5
18804	1800	4	4	4	4
18840	200	3	3	4	4
19029	600	4	4	4	5
19183	1800	4	5	4	3
19720	330	4	4	4	3
19838	1356	4		5	4
20044	1739	4	4	4	5
20089	288	3		4	4
20143	135	3	3	3	4
20183	2090	5	4	3	4
20435	2200	5	5	5	
20444	243	3	4	4	4
20540	9000	6	6	6	3
20682	2500	5	5		4
20733	1900	4	4	4	6
20911	2100	5	3		3
20950	2.54	1	2	4	5
20977	1000	4	4		4
21009	500	4	4	4	3

Compound ID	LD50 (mg/kg)	Toxicity class	ProTox – predicted class	Topkat® - predicted class	T.E.S.T. – predicted class
21136	330	4	4	4	4
21295	10000	6		4	6
21324	23	2	2	3	
21343	620	4	4	4	4
21417	1700	4	4	6	4
21472	1000	4	5		4
21488	370	4	5	4	
21501	2000	4	5		4
21700	90	3		3	3
21794	6000	6	6	5	4
21967	2759	5	4	5	4
21969	678	4	4	5	4
22048	2550	5	2		3
22160	5000	5	2	4	4
22380	500	4	4	4	3
22410	3000	5	5	3	3
22524	180	3	3		
22529	3125	5	5	4	
22641	476	4	4	6	4
22666	300	3		4	4
22746	200	3	3	4	4
22802	7500	6	3	5	6
22929	400	4	4	4	4
23016	22	2	3	4	5
23038	2000	4	4	5	4
23156	98	3	3	4	5
23182	800	4	4		4
23426	1000	4	5	5	3
23477	1213	4	4	4	4
23754	1800	4	4	3	5
23887	2240	5	5	3	4
23962	6.55	2	1	5	5
24014	1570	4	5	4	5
24042	75	3	3	6	4
24163	1540	4		4	3
24251	0.8	1	1	3	2
24347	250	3	3	4	4
24355	2125	5	4	4	5
24426	20	2	2	3	3
24449	810	4	4	4	2
24542	1000	4	4	4	4
24543	200	3	3	6	4
24546	1000	4		4	4
24741	227	3	4	3	4
24777	1300	4		4	
24854	2400	5	5		6
24884	28	2	4	4	4
25031	305	4	3	4	6
25119	2800	5	6		6
25157	10000	6	6	3	5
25169	2500	5	4		4

Compound ID	LD50 (mg/kg)	Toxicity class	ProTox – predicted class	Topkat® - predicted class	T.E.S.T. – predicted class
25498	27	2		6	
25622	609	4		3	4
25626	1000	4	4	4	5
25671	538	4	4	4	4
25701	616	4	3		2
25709	50	2		4	
25714	500	4	4	3	4
25733	347	4	3	4	4
25818	3000	5	4	4	3
25845	250	3	3	4	4
25888	371	4			5
25933	3.32	1	1	4	4
25975	760	4	4	4	4
25977	310	4	4	3	4
26032	1480	4	4	3	
26057	560	4	3	4	5
26070	410	4	3	4	4
26172	1309	4	5	4	6
26233	824	4	4	4	4
26240	700	4	4	4	4
26519	4350	5	5	4	6
26764	500	4	4		3
26855	335	4	4	3	4
26870	5200	6			
26904	511	4	4	4	4
26938	8.05	2	2	4	4
26994	48.5	2	4	3	2
27095	264	3	3	4	4
27160	220	3	3	2	2
27280	800	4	4	5	4
27294	260	3	4	3	3
27475	1250	4	4	3	
27594	150	3	1	5	3
27647	2000	4	4	4	3
27663	50.1	3		4	2
27734	1000	4		4	5
27857	1000	4	4	5	5
27861	74	3		3	2
27988	660	4	3	3	5
28079	1500	4	4	4	4
28136	550	4	3	4	4
28153	178	3	3	3	3
28166	740	4	4	3	3
28351	3750	5	4	3	3
28396	1100	4	4	5	4
28468	1000	4	5	6	6
28565	2000	4	4	3	4
28572	1100	4	4	4	6
28614	890	4	4	3	5
28688	700	4	4	4	4
28766	1092	4		5	3

Compound ID	LD50 (mg/kg)	Toxicity class	ProTox – predicted class	Topkat® - predicted class	T.E.S.T. – predicted class
28841	500	4	4		3
28846	1500	4			
28982	700	4	4	6	6
29039	395	4	4	5	4
29108	4920	5	5	4	6
29177	2750	5	4	4	5
29214	8.2	2	1	5	4
29229	4000	5	4		
29342	1000	4	4	4	4
29374	16.9	2		4	3
29471	122	3	3	4	4
29584	500	4	4	4	5
29614	10000	6	6	4	3
29618	319	4	4	3	4
29622	500	4	4	4	4
29822	190	3	3	5	3
29857	1000	4	4	3	2
29924	4650	5	5	4	5
30087	1000	4	4	4	6
30164	14	2	6	5	6
30337	15.2	2	2	3	4
30348	1475	4	4	4	4
30413	1415	4		4	3
30547	1832	4	4	4	5
30578	2000	4	4	4	6
30636	1500	4	4	4	
30740	1850	4	5	5	6
30760	1000	4	4	3	5
30780	1300	4		4	6
30947	2000	4	4	5	6
31018	600	4	4	4	5
31086	580	4	2	6	6
31108	1300	4		4	4
31130	23500	6	6		6
31460	5000	5		5	5
31483	4000	5	5	4	4
31526	1820	4	4	4	4
31539	2817	5	5		4
31547	74	3	3	3	
31575	3000	5	5	4	2
31781	195	3	4	4	4
31802	5000	5	5	4	5
31818	35	2	2	4	4
32074	590	4	4	4	4
32162	2450	5	5	4	5
32171	1000	4	4	3	4
32253	15.8	2	2	4	2
32543	2300	5	5	4	4
32613	1235	4	4	3	4
32724	10000	6		3	
32858	1800	4	4	3	3

Compound ID	LD50 (mg/kg)	Toxicity class	ProTox – predicted class	Topkat® - predicted class	T.E.S.T. – predicted class
33038	214	3	3	4	4
33148	7500	6	6	4	6
33236	500	4	3	5	4
33261	706	4	4	4	4
33319	2000	4	4	3	4
33341	530	4			
33571	5000	5	5	4	6
33660	5000	5	5	5	4
33699	1725	4	4	4	4
33845	642	4	4	4	4
33944	1000	4	4	4	3
33975	390	4	4	3	4
34065	250	3	4	4	3
34139	2000	4	4	6	4
34155	1000	4	4	4	3
34310	280	3	3	4	3
34318	21000	6	4	4	5
34587	500	4	4	3	2
34680	1200	4	4	3	4
34764	10000	6	3	4	4
35076	1100	4	4	4	4
35103	1200	4	4	6	4
35246	4700	5	5	4	5
35268	1410	4	5	5	4
35538	3000	5		4	4
35553	800	4		4	4
35724	800	4	4	4	4
35732	2000	4	4		3
35738	500	4	4	4	4
35810	51.87	3	2		2
35876	4100	5		4	
35879	500	4	4	5	3
35975	1320	4	4	4	5
35993	2100	5	4	5	5
36010	647	4	4	4	4
36055	2765	5	4	4	4
36057	711	4		4	5
36152	760	4	4	3	5
36360	145	3	3	4	4
36419	660	4	4	4	4
36459	670	4	4	3	5
36472	2000	4	2		2
36490	450	4	4	4	4
36610	475	4	4	4	4
36639	3.3	1	2	3	3
36666	9.66	2	2		2
36710	600	4	4		4
36718	770	4	4	4	4
36799	9	2		6	6
36898	45	2	3	4	4
37242	560	4	3	3	3

Compound ID	LD50 (mg/kg)	Toxicity class	ProTox – predicted class	Topkat® - predicted class	T.E.S.T. – predicted class
37271	270	3	3	4	
37287	1000	4	4		3
37539	2150	5	5	4	4
37720	50	2	2	3	4
37725	2125	5	5	4	4
37862	170	3	3	3	5
37880	310	4	4	4	3
37931	1000	4	4	4	2
37985	750	4	4	4	4
38143	600	4	4	4	4
38152	1500	4		4	4
38155	638	4	4	3	4
38227	4490	5	6	4	5
38336	2500	5	4	4	5
38342	2300	5	5	5	6
38400	3750	5	5	5	5
38406	2161	5	6	5	5
38434	2000	4	4	4	6
38744	2991	5	5	5	4
38752	500	4	4	4	5
38768	15000	6		3	5
38817	3000	5	5	2	2
38823	620	4	4	4	4
38938	325	4	4	5	4
38974	345	4		6	3
39021	173	3	3	4	4
39079	318	4	4	4	2
39101	560	4	4	4	5
39227	5000	5	5	6	5
39301	849	4	4	5	4
39350	110	3	4	5	4
39404	1000	4	4	4	4
39413	700	4	3	4	6
39565	27.79	2	2	3	6
39581	2070	5	5	5	5
39773	125	3	3	4	4
Sensitivity (%)<br>Specificity (%)<br>Precision (%)<br>Coverage (%)			72.49<br>94.50<br>72.68<br>85.80	42.41<br>88.48<br>42.56<br>89.60	40.70<br>88.14<br>43.45<br>91.40

The performance was analyzed based on a set of 500 diverse molecules The set consist of diverse compounds belonging to different toxicity classes and showing a toxicity class distribution similar to the dataset used for prediction. Diverse molecules were identified using the search for diverse sets using ECFP4 fingerprints as implemented in the Discovery Studio 3.1 software (Accelrys Inc, USA).

[1] ProTox similarity search using FP24 fingerprints and toxic fragments; [2] TOPKAT® (Accelrys Inc, USA) oral rat LD_{50} model; [3] TEST (USA) oral rat LD_{50} model using nearest neighbor prediction

S4 Validation of toxicity target pharmacophores

Toxicity target	Abbreviation (Uniprot name)	Pharmacophores with AUC > 06	AUC of average fit
Adenosine A2a receptor	AA2AR	2	0.68
Adrenergic beta 2 receptor	ADRB2	7	0.87
Androgen receptor	ANDR	45	0.88
Amine oxidase A	AOFA	3	0.70
Corticotropin-releasing hormone receptor 1	CRFR1	0	N/A
Dopamine D3 receptor	DRD3	1	0.61
Estrogen receptor 1	ESR1	192	0.95
Estrogen receptor 2	ESR2	32	0.96
Glucocorticoid receptor	GCR	7	0.84
Histamine H1 receptor	HRH1	1	0.96
Nuclear receptor subfamily 1 group I member 2	NR1I2	97	0.81
Opiod receptor kappa 1	OPRK	4	0.80
Phosphodiesterase 4D	PDE4D	152	0.73
Prostaglandin G/H synthase 1	PGH1	1	0.66
Progesterone receptor	PRGR	9	0.79
